# Flowering Coriander (*Coriandrum sativum*) Strips Do Not Enhance Ecosystem Services in Azorean Orchards

**DOI:** 10.3390/insects14070634

**Published:** 2023-07-14

**Authors:** Marco Ferrante, Gabor L. Lövei, Lambert Lavigne, Mario Caballero Vicente, Elisa Tarantino, David Horta Lopes, Paulo Monjardino, Paulo A. V. Borges

**Affiliations:** 1 cE3c-Centre for Ecology, Evolution and Environmental Changes, Azorean Biodiversity Group, CHANGE–Global Change and Sustainability Institute, Faculty of Agricultural Sciences and Environment, University of the Azores, PT-9700-042 Angra do Heroísmo, Portugal; marco.ferrante@uni-goettingen.de (M.F.);; 2 Functional Agrobiodiversity, Department of Crop Science, Georg-August University of Gottingen, DE-37077 Gottingen, Germany; 3 Flakkebjerg Research Centre, Department of Agroecology, Aarhus University, DK-4200 Slagelse, Denmark; 4 ELKH-DE Anthropocene Ecology Research Group, Debrecen University, HU-4032 Debrecen, Hungary; 5 CBA–Biotechnology Centre of Azores, Faculty of Agricultural Sciences and Environment, University of the Azores, PT-9700-042 Angra do Heroísmo, Portugal

**Keywords:** agro-environmental scheme, ecological intensification, ecosystem disservice, ecosystem function, sustainable agriculture

## Abstract

**Simple Summary:**

Ecosystem services (ESs) and disservices (EDs) are routinely estimated from changes in service provider densities without measuring their actual levels. By using the sentinel approach (i.e., exposing a plant, seeds, and prey models in a standardized way), we tested how coriander (*Coriandrum sativum*) strips planted in mixed orchards on Terceira Island (Azores, Portugal) affected ESs/EDs via herbivory on lettuce plants, seed predation on wheat and weed seeds, and predation on artificial caterpillars. Vertebrates had more influence than invertebrates on ESs and EDs. Herbivory (ED) after 2 weeks was similar in the coriander and the control plots, while seed predation was higher in the control than in the coriander plots on both wheat grain (an ED: 30.8% vs. 15.3%) and weed seeds (an ES: 2.5% vs. 0.4%). Vertebrate predation (ES) rates after 48 h were significantly higher in the control (9%) than in the coriander plots (3%), while no difference was observed for invertebrate predation. Coriander strips did not support increased ES/reduced ED levels in this habitat. The sentinel approach is effective to quantitatively compare multiple ESs/EDs under different farming management strategies.

**Abstract:**

The effect of flower strips on ecosystem services (ESs) and disservices (EDs) is routinely assessed following changes in service provider densities without measuring the associated levels of ES/EDs. By using the sentinel approach (i.e., exposing a plant, seeds, and prey models in a standardized way), we tested how coriander (*Coriandrum sativum*) strips planted in mixed orchards on Terceira Island (Azores, Portugal) affected herbivory on lettuce plants, seed predation on wheat and weed seeds, and predation on artificial caterpillars. Vertebrates had more influence than invertebrates on ESs/EDs. Herbivory (ED) after 2 weeks was similar in the coriander and the control plots (mean ± SD; 2.3% ± 3.3% vs. 2.2% ± 2.9%, n = 32 for both). Seed predation was higher in the control than in the coriander plots for both grain (ED; 30.8% ± 38.9% vs. 15.3% ± 10.8%, n = 18 for both) and weed seeds (ES; 2.5% ± 4.1% vs. 0.4% ± 0.5%, n = 18 for both). Vertebrate predation (ES) rates after 48 h were significantly higher in the control (estimate 9%, 95% CI: 4–20%) than in the coriander plots (3%, 1–8%), while no difference was observed for invertebrate predation. Coriander strips did not support increased ES/reduced ED levels in this setting. The tools used can be effective to quantitatively compare multiple ESs/EDs under different farming management strategies.

## 1. Introduction

In cultivated landscapes, biodiversity provides numerous ecosystem services (ESs), such as pollination, and pest and weed control, as well as ecosystem disservices (EDs, [1]), such as herbivory and intraguild predation. Modern agriculture typically creates monocultures, and relies on chemical inputs and continued intervention, causing biodiversity loss and an alarming decrease in arthropod biomass [2]. Alternative management strategies have been suggested to make the agroecosystem more supportive of local biodiversity, with the hope of boosting ESs (e.g., ecological intensification [3]). The effectiveness of such measures is routinely tracked by monitoring the abundance or diversity of the identified ES providers, which does not always reflect the levels of ESs [4,5]. A confounding factor is that a species cannot always be unequivocally labeled beneficial or harmful, as the same can be an ES provider and, at other times, an ED provider [6]. For instance, ground beetles (Coleoptera, Carabidae) in strawberry fields can act as natural enemies and crop pests and be both ES and ED providers [7]. Adult Lepidoptera can contribute to pollination, while their larvae can be crop pests. Instead of using proxies (e.g., ES provider abundance or diversity), the levels of ESs can be directly measured using standardized techniques. However, this is rarely applied or is restricted to singular ESs (e.g., [8]). 

The establishment of flower strips at field margins or within cultivated fields is intended to boost local biodiversity, especially of beneficial arthropods [9]. Flower strips sustain biodiversity (e.g., pollinators and natural enemies) by providing food, refuge, overwintering and oviposition sites, and offering favorable microclimatic conditions [10]. The benefits of flowering strips have been documented in several situations including orchards [11], broadacre crops [12], and in temperate [13] and tropical [14] areas. However, their effect has been mostly tracked by observing or collecting flower-visiting insects. Most studies measured ESs indirectly, using proxies (e.g., [15]); when ESs are directly measured, usually only single ESs are considered and EDs are neglected [16]. Therefore, whether the presence of flower strips translates to higher levels of ESs is unclear, and the overall impact of flower strips on ESs (and EDs) remains uncertain.

Flower strips can be spontaneous [17] or a precomposed mix [18] but they usually contain species that may be considered weeds, and this could hamper their acceptance by farmers. To try to circumvent this potential problem, we decided to use a minor crop, coriander (*Coriandrum sativum* L.; Magnoliopsida, Apiales, Apiaceae), which is often included in flower strips to attract natural enemies and pollinators [19,20,21]. Additionally, we used a single species to see if such strips could prove useful because some of the benefits emerge from habitat structure rather than the identity of the flowering plant [10]. We assumed that farmers would more easily accept this method if it was (a) composed of a single species rather than a mix, (b) a commercial plant rather than one that might be considered a weed, and (c) not a potentially invasive species. 

The effectiveness of coriander strips to enhance ESs and their effect on selected EDs were assessed using the sentinel approach, which is suitable to obtain quantitative, comparable data from different habitats [22]. This approach relies on the repeatability of standardized monitoring tools (i.e., sentinels). Sentinels can be real or artificial models that represent or emulate a resource (e.g., a prey, a seed, or a plant) [22]. Although sentinels may not imitate locally occurring species, they are useful in comparative studies to measure the intensity of selected ecological processes in different setups. Here, we quantified herbivory on a crop plant (ED), seed predation on a grain (ED) and on a weed seed (ES), and predation pressure on invertebrates (ES). We left pollination out of consideration on purpose, because the citrus trees produce plenty of attractive flowers; hence, planting more flowers would not make a large difference for this function.

As habitat heterogeneity usually boosts biodiversity [23], and coriander attracts beneficial arthropods, we hypothesized that, in the plots with the coriander strips, the levels of all ESs would be higher while that of EDs would be lower than in the control plots. We also hypothesized that the highest differences would be observed during coriander flowering when pollen and nectar are available, which can attract flower-visiting arthropods [24].

## 2. Materials and Methods

### 2.1. Study Orchards

Our study orchards were located on Terceira Island, a geologically young volcanic island of the Azores archipelago in the North Atlantic Ocean. The Azores belong to the Macaronesian biome [25] and have an oceanic climate (750–1700 mm annual rainfall, 13.8 °C in February and 22.3 °C in August). The original vegetation consists of laurisilva forests [26], which currently occupy <5% of their original area, as they were cleared to expand pastures and croplands since the discovery of the archipelago [27]. Today, agricultural areas dominate on Terceira [28], mostly composed of intensively managed pastures, maize fields, mixed orchards, and vineyards.

This study took place near the city of Angra do Heroísmo in three mixed orchards (sized between 5.7 ha and 11.3 ha) of comparable age and similar fruit tree composition: dominated by orange (*Citrus × sinensis* Osbeck) and banana (*Musa* sp.), with fewer chestnut (*Castanea sativa* Mill), loquat (*Rhaphiolepis japonica* (Lour.) Galasso & Banfi), and yellow guava (*Psidium guajava* L.) trees. Non-crop shrubs, such as the exotic *Pittosporum undulatum* Vent. and *Banksia integrifolia* L.f., and the endemic *Morella faya* (Aiton) Wilbur formed hedgerows. The three orchards (Quinta do Rosario: 38°40′57.7″ N, 27°15′46.1″ W; Bicas: 38°40′15.9″ N, 27°14′30.4″ W; San Bartolomeu: 38°40′51.1″ N, 27°16′33.7″ W) were located between 1 km and 3 km from each other and surrounded by a similar peri-urban landscape. 

### 2.2. Experimental Design

In each of these orchards, one coriander strip 5 m long and 1.5 m wide was established in the center of a parcel of approximately 30 × 20 m (hereafter “coriander plot”), and one control area with a low, spontaneous grass undergrowth was selected at least 100 m away (hereafter “control plot”). All orchards received no herbicide or insecticide applications during the study. Coriander strips (2.5 m × 1.5 m) were sown on 2 February 2021 (Quinta do Rosario and Bicas) and 3 February 2021 (San Bartolomeu) at a 40 kg/ha rate (Figure S1). After 2 weeks, an additional 2.5 m × 1.5 m strip was added to extend the period when coriander flowers were present. On 29 March 2021, all coriander strips were reseeded at a 2 g/m^2^ rate to fill gaps. No other herbaceous flowering plants were present in large numbers in our study orchards. A molluscicide bait (active ingredient metaldehyde) was applied at sowing. The ecological process assessments were performed before (April), during (June), and after (July) coriander flowering. 

### 2.3. Measuring Levels of Herbivory

Lettuce (*Lactuca sativa* L.) plants were selected as sentinels for characterizing herbivory because several potential herbivores will willingly consume them [29]. Sentinel plants (cv. Lirice, Vilmorin seeds, La Ménitré, France) were grown in 5 L pots in the greenhouse at the University of the Azores, Angra do Heroísmo campus. Plants were regularly watered and brought to the orchard when they reached the full head formation stage (i.e., commercial size). In the orchard, the pots were dug into the soil at the four corners of the coriander and grass strips (Figure S2) where they remained exposed to herbivores for 2 weeks (Figure S3). After exposure, herbivore damage was visually assessed on individual leaves following Johnson et al. [30]. We only measured leaf loss on all fully developed leaves (>10 cm in length) as they do not rapidly grow; thus, their loss of leaf surface would better reflect the actual rate of herbivory than the same on small, rapidly growing leaves. Overall, we used 24 lettuce plants (4 plants per plot × 2 treatments × 3 orchards) for each sampling event. Herbivory was measured during 13–27 April 2021 (before coriander flowering), 5–19 June 2021 (during flowering), and 28 June–11 July 2021 (after flowering).

### 2.4. Assessing the Levels of Seed Predation

Sentinels for measuring seed predation included seeds of mustard (*Sinapis alba* L.), representing a common weed [31], and seeds of wheat (*Triticum aestivum* L.), the predation on which constitutes an ED. These species were selected because they are commercially available and consumed by seed predators [32,33]. Seed predation by invertebrates and vertebrates was measured using modified food storage containers (Tupperware, Orlando, FL, USA) covered with a lid to avoid rain and interference by birds [34]. In each seed box (15 cm × 15 cm × 9 cm), we first placed 50 of either mustard seeds or wheat grains in a grid of 5 columns × 10 rows using a double-faced adhesive strip, which was subsequently sprinkled with sifted soil to allow seed predators to explore the boxes without getting stuck. Half of the boxes were made inaccessible to larger animals by gluing a 1 cm × 1 cm mesh over the openings of the boxes (Figure S4). This way, we were able to distinguish between the impact of all vs. invertebrate seed predators. One replicate consisted of a group of four boxes: one open and one vertebrate exclusion box with mustard seeds, and a similar pair with wheat grains. The four boxes were arranged in a square, 1 m from each other (Figure S2). On each sampling occasion and in each orchard, we exposed four groups at the corners of the coriander and the control strips, yielding a total of 96 seed boxes per sampling event (4 boxes × 2 box types × 2 seed types × 2 treatments × 3 orchards) and 288 during the experiment. Seed boxes were exposed for 48 h, after which we recorded the number of seeds that were damaged or disappeared (Figure S5). Seed predation was measured on 14–16 April 2021, on 7–9 June 2021, and in early July (8–10 July 2021 at Bicas and San Bartolomeu, 11–13 July 2021 at Quinta do Rosario). 

### 2.5. Assessing Predation Pressure

Predation on invertebrates was measured using the artificial caterpillar method [35] (Figure S6). This method records the activity of vertebrate and invertebrate predators that are identified by the characteristic marks left on the plasticine prey after an attack. Caterpillars were made of green plasticine (Smeedi plus, V. nr. 776609, Denmark), 15 mm long and 3 mm diameter. The green color was selected because it is typical of palatable prey that are not chemically defended, while such dimensions were chosen because they are close to the real size of caterpillars [36]. In each orchard, in the coriander and control plots, three groups of 10 caterpillars were exposed on 14–16 April 2021, while five groups of 10 caterpillars were exposed on 7–9 June 2021, 8–10 July 2021 (Bicas and San Bartolomeu), or 11–13 July 2021 (Quinta do Rosario). Groups were at least 1.5 m from each other. Each group was made of two parallel lines of five caterpillars 1 m from each other (Figure S2). When only three groups of 10 caterpillars were used, we selected those closer to the coriander strip. We used 180 caterpillars in April 2021 (30 caterpillars × 3 orchards × 2 treatments) and 300 caterpillars (50 caterpillars × 3 orchards × 2 treatments) in June and July 2021 for a total of 780 caterpillars. Lost caterpillars (n = 21, 2.7%) were not considered predated and were excluded from the analysis. Attack marks were identified by the first author, on the basis of previous personal experience [37] and Low et al. [38].

### 2.6. Statistical Analysis

All statistical analyses were performed using R version 4.1.1 [39] through RStudio [40]. The R packages *performance* [41], *DHARMa* [42], and *ggeffects* [43] were used to validate and visualize the statistical models. Post hoc tests were performed using the *lsmeans* package [44]. Except for the wheat seed predation model, we did not include the interaction between treatment and coriander phenological stage in the final models, as it was not significant. Significances were confirmed using the Anova function in the package *car* [45] to test for type II and III ANOVAs. An overview of the models can be found in Table S1, and datasets are available in Data Availability Statement.

#### 2.6.1. Analyzing Herbivory

After calculating average herbivory on each sentinel lettuce, herbivory damage was analyzed in a zero-inflated beta-regression mixed model with damage as the response, the treatment (coriander vs. control) and the coriander phenology (before, during, and after flowering) as additive fixed factors, and orchard ID as the random factor.

#### 2.6.2. Analyzing Seed Predation

Seeds that were damaged or disappeared were considered predated. As seed predation is a locally variable phenomenon [46], we analyzed both the percentage of seed boxes predated and the predation rate (percentage of seeds predated).

We tested whether the probability that at least one seed in a seed box was predated using a generalized linear mixed model (GLMM) with binomial distribution and logit link function. The occurrence of predation (yes/no) was the model response; the treatment (coriander vs. control), the coriander phenology (before, during, or after flowering), the seed species (wheat vs. mustard), and the box type (open vs. vertebrate exclusion) were the fixed factors; the box ID and the orchard were nested random factors.

Wheat and mustard seed predation rates were analyzed using two separate GLMMs with zero-inflated beta binomial distribution where the seed predation was the response, the treatment, the coriander phenology, and the box type were the fixed factors, and the box ID and the orchard were nested random factors. Since box type was not significant, this factor was removed from the final models (Table S1).

#### 2.6.3. Analyzing Predation

Predation rates by invertebrates and vertebrates were analyzed separately because the same factors can affect these two predator groups in different directions, and complex responses may be overlooked when analyzing the total predation [47]. We constructed two GLMMs with binomial distribution and logit link function. The model included predation (yes/no) as response, treatment and phenology as fixed factors, and the orchard and group of caterpillars as nested random factors.

## 3. Results

### 3.1. Herbivory

**Figure 1:**
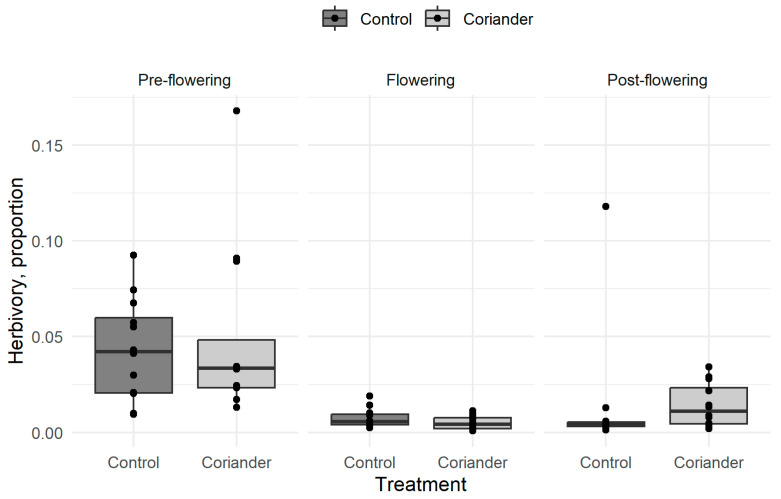
Herbivory rates in coriander and control strips in Azorean orchards. The thick black line corresponds to the median, the lower and the upper sides of the box indicate the upper and lower quartiles, respectively, and the whiskers extend to 1.5× the interquartile range. Points are observed means.

The average leaf surface loss on a lettuce plant after 2 weeks was 2.2% (SD = 3.0%, n = 72). Herbivory was not significantly different between the coriander and the control plots but significantly higher before than during or after coriander flowering, and after flowering than during flowering (Figure 1; Table 1, Tables S2 and S3). We did not try to identify herbivores responsible for the damage, but we occasionally observed slugs, snails, and caterpillars on the sentinel plants.

### 3.2. Seed Predation

**Figure 2:**
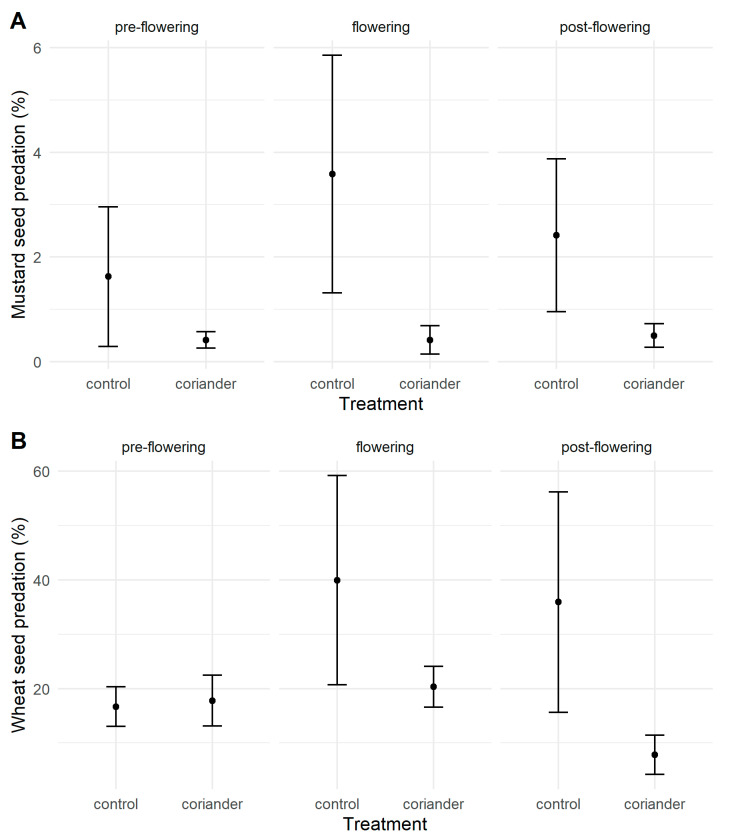
Mustard (**A**) and wheat (**B**) seed predation rates in coriander and control strips in Azorean orchards. Points are means; vertical lines indicate ± SE.

More than half of the boxes (51.0%) suffered no seed predation, while 17 of them (5.9%) had 100% predation (16 at Quinta do Rosario, all with wheat grains). We registered intermediate levels of seed predation (43.1%) in the rest of the boxes. The probability that at least one seed in a box was predated was significantly higher with wheat than with mustard seeds and in the control than in the coriander plots, irrespective of the seed predators or the coriander phenology (Tables S4 and S5). Most of the predated seeds (61.1%) disappeared, while the rest were damaged. Wheat seed predation was not significantly different in the treatment and control plots before coriander flowering, but it was significantly higher in the control plots than in the coriander plots both during and after flowering (Figure 2, Table 1, Tables S6 and S7). Mustard seed predation was significantly higher in the control than in the coriander plots and during coriander flowering than before and after it (Figure 2, Table 1, Tables S8 and S9).

### 3.3. Predation

**Figure 3:**
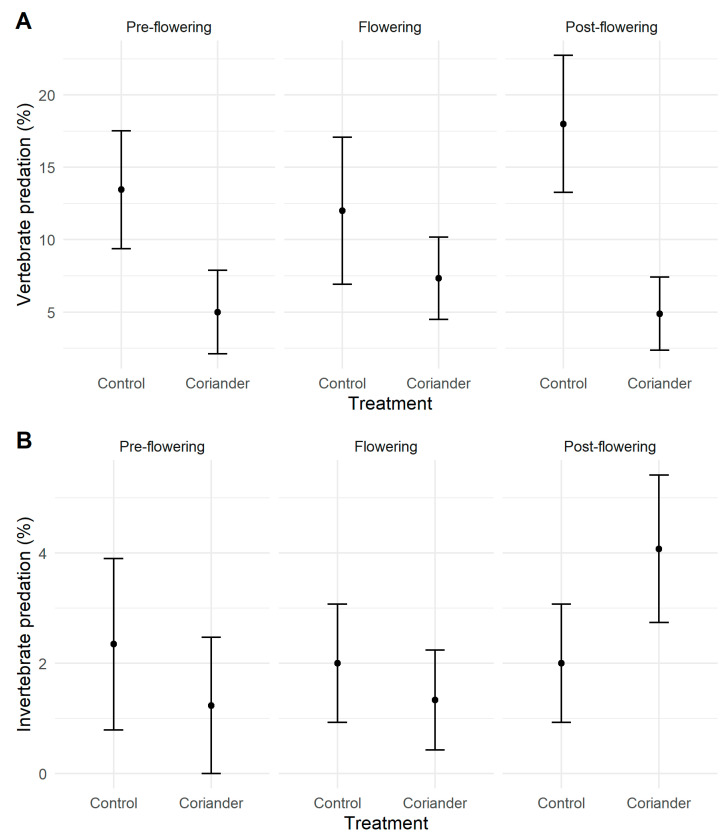
Vertebrate (**A**) and invertebrate (**B**) predation rates in coriander and control strips in Azorean orchards. Points are means; vertical lines indicate ± SE.

Of the 759 caterpillars exposed and recovered, 95 (12.5%) were attacked after 48 h. Rodents were responsible for 52.6% of the attacks, along with birds for 29.5% and arthropods for 17.9%. Mammal predation rates were higher at Quinta do Rosario than the other two locations, while bird predation rates were highest at San Bartolomeu. Arthropod predation rates were lower than vertebrate ones and similar in all studied orchards (Figure S7). Vertebrate predation rates were significantly higher in the control than the coriander plots. However, no significant differences were registered before, during, and after flowering (Figure 3, Table 1, Tables S10 and S11). Arthropod predation rates were the highest after flowering (Table 1). Neither the treatment nor phenology significantly affected invertebrate predation (Figure 3, Tables S10 and S11).

## 4. Discussion

### 4.1. ESs and EDs in Azorean Mixed Orchards

In this study, we focused on herbivory, seed predation, and predation on caterpillars, and found that coriander strips did not enhance these processes. The levels of ESs and EDs we obtained were generally lower in our island orchards than what was found in mainland temperate agroecosystems. However, comparisons are hampered by the lack of a standardized methodology, particularly for herbivory. Herbivory is commonly estimated by measuring the missing leaf area [48]. This approach, however, often provides imprecise data because the time frame during which the damage occurred is unknown [49]. The herbivory rate we registered after 2 weeks corresponds to a daily loss of 0.16%. This is slightly lower than what we observed in other Azorean orchards in 2020 (daily loss of 0.26%), but higher than the herbivory rates recorded in vineyards (daily loss of 0.04%) using the same methodology [22]. Contrary to our hypothesis, the highest herbivory rates were recorded in spring before coriander flowering, and it is possible that this pattern was due to the higher activity of slugs and snails, the main herbivores detected, during the wetter spring months.

Seed predation was especially high on wheat seeds (11.3% per day), suggesting that large seed predators, likely rodents, which are exotic to the Azores, are important ED providers. These results are consistent with observations in Sweden using wheat and hemp-nettle seeds [32,50]. Rodents are well-known and often abundant invaders on oceanic islands, where they threaten local biodiversity [51]. It is likely that the low seed predation rates recorded on mustard seeds (0.75% per day) occurred due to the species-poor seed predator community on Terceira Island. Carabids can be important seed predators [52] but only two such species, *Pseudoophonus rufipes* (De Geer, 1774) and *Laemostenus complanatus* (Dejean, 1828), are relatively common in Azorean orchards [53], and only the former is known to consume seeds. Similarly, of the five species of ants occurring on Terceira, only *Lasius grandis* Forel (1909) and *Tetramorium caespitum* L. (1758) are abundant, and neither species harvests seeds. Therefore, despite the high protein content of mustard seeds [54], this resource remained largely unexploited, indicating that weed seed predation rates may be limited by a lack of suitable ES providers. The fact that more than half of the seed boxes showed no predation, while some were completely depleted of seeds, confirms that seed predation is a locally variable phenomenon [46].

The predation rate on an artificial caterpillar was 6.3% per day. This was much lower than recorded in orchards in tropical countries. In an Indian forest bordering an orchard, 76% of the artificial prey were attacked after 48 h [55]. In tea plantations in southeastern China, 21.9% of the artificial prey were attacked after 24 h [56]. In Malaysian orchards, 31% of the artificial prey were attacked after 72 h [57]. Compared to these studies, arthropods provided a smaller contribution to predation in Azorean orchards, while rodents and birds were relatively more important. The documented absence of large predatory ground beetles, such as *Carabus* spp. or *Pterostichus* spp., from Azorean orchards [58] could be responsible for this difference.

### 4.2. The Effectiveness of Coriander Strips

Previous studies have shown the potential of intercropping with coriander to attract natural enemies [21,59,60]. We found that, on Terceira Island, vertebrate service providers were more influential than invertebrates, but our hypothesis of increased ES/reduced ED levels by coriander strips, even during flowering, was not supported. This can be caused by several, non-mutually exclusive reasons.

Due to their isolation, oceanic islands have a more limited species pool than “mainlands”, and the guilds that can be attracted by such habitat manipulation may be less species-rich and of lower abundance. This known ecological pattern could explain why the levels of ESs/EDs we measured were generally lower than what was found on various continents using the same or similar methods. A recent survey found only 122 species in mixed orchards on Terceira Island, including well-known ES providers such as spiders (22 spp.), rove beetles (20 spp.), ground beetles (seven spp.), and ants (five spp.; [53]). Arthropod species richness in similar European mainland habitats is much higher [61].

At a smaller scale, our study orchards were also isolated from the native forests, which, on Terceira, is restricted at the higher elevations (i.e., over 500 m above sea level). It is possible that isolation from the natural habitats hampers the immigration of beneficial arthropods into orchards.

Enhancing crop diversity provides resources for beneficial arthropods, and the diversity of chemical cues may confuse herbivores [62]. However, ecological theory suggests that interventions such as establishing flower strips should be more effective in simple than complex landscapes [63]. The Azorean orchards are polycultures with complex horizontal and vertical stratification. Adding an extra plant species to the already rich plant species pool may not have been as effective as in a less complex and less diverse habitat.

In this study, we aimed to sample intensively and directly multiple ecological processes to demonstrate the suitability of the sentinel approach, which limited the number of orchards to be involved. While the importance of ESs is being recognized, the status of most ESs keeps worsening [64,65], and leaving out the effect on Eds of interventions that intend to boost ESs may undermine their effectiveness. We argue that including direct measurements of both ESs and EDs may usefully complement the evaluation of interventions intended to improve agricultural sustainability. Island agriculture may require a wider-scope evaluation of environmental impact than mainland practices, and the methodology we used here may provide a tool for that aim.

## Figures and Tables

**Table 1:** Rates (mean percentage ± SD) of ecosystem functions in coriander and control plots in Azorean orchards before, during, and after coriander flowering.

		Rate		
Ecosystem Function	Phenology	Coriander	Control	n
Herbivory	Pre-flowering	4.35 ± 2.66	4.88 ± 4.54	12
	Flowering	0.76 ± 0.5	0.52 ± 0.38	12
	Post-flowering	1.4 ± 3.29	1.42 ± 1.13	12
Seed predation, open, wheat	Pre-flowering	20.43 ± 14.67	16.5 ± 5.89	3
	Flowering	17.67 ± 12.39	43 ± 50.09	3
	Post-flowering	8.83 ± 12.29	35.17 ± 56.15	3
Seed predation, open, mustard	Pre-flowering	0.5 ± 0.5	2.73 ± 4.72	3
	Flowering	0.33 ± 0.58	3.67 ± 6.35	3
	Post-flowering	0.83 ± 0.58	3.83 ± 5.01	3
Seed predation, exclusion, wheat	Pre-flowering	15.17 ± 9.44	16.83 ± 12.86	3
	Flowering	23 ± 6.06	36.83 ± 54.87	3
	Post-flowering	6.83 ± 6.53	36.67 ± 54.85	3
Seed predation, exclusion, mustard	Pre-flowering	0.33 ± 0.29	0.5 ± 0.5	3
	Flowering	0.5 ± 0.87	3.5 ± 6.06	3
	Post-flowering	0.17 ± 2.89	1 ± 0.87	3
Vertebrate predation	Pre-flowering	5 ± 8.57	12.46 ± 12.22	9
	Flowering	7.3 ± 11	12 ± 19.71	15
	Post-flowering	4.89 ± 9.83	18 ± 18.33	15
*Invertebrate predation*	Pre-flowering	1.23 ± 3.7	2.35 ± 4.67	9
	Flowering	1.33 ± 3.52	2 ± 4.14	15
	Post-flowering	4.07 ± 5.17	2 ± 4.14	15

## Data Availability

Data will be made available on reasonable request.

## Supplementary Materials

The following supporting information can be downloaded at https://www.mdpi.com/article/10.3390/insects14070634/s1: Figure S1. Coriander (*Coriandrum sativum*) strip starting to flower in a mixed orchard on Terceira Island, Azores, Portugal; Figure S2. Arrangement of the sentinels in the orchards. The green circles represent individual sentinel lettuce plants; each orange square represents a group of four seed boxes (open and vertebrate exclusion box with mustard seeds, and open and vertebrate exclusion box with wheat grains) arranged in a square, 1 m from each other; the green lines represent a transect consisting of five artificial caterpillars linearly arranged, 1 m from each other; Figure S3. Sentinel lettuce (*Lactuca sativa*) near one of the corners of the coriander strip in a mixed orchard on Terceira Island, Azores, Portugal; Figure S4. An “open” box accessible to both invertebrates and vertebrates (on the left) and a vertebrate exclusion box (on the right). Seed boxes were covered with a lid to avoid rain and interference by birds; Figure S5. Wheat seeds drilled by seed predators inside a vertebrate exclusion box in a mixed orchard on Terceira Island, Azores, Portugal; Figure S6. Artificial caterpillar made of plasticine glued to a piece of reed and exposed at ground level in a mixed orchard on Terceira Island, Azores, Portugal. The artificial caterpillar has been attacked by a bird, which can be identified by the typical “v” mark; Figure S7. Relative predation by birds, invertebrates, and mammals in three Azorean orchards; Table S1. Model overview; Table S2. Herbivory model output; Table S3. The results of the Lsmeans test on the herbivory model; Table S4. Seed box predation model output; Table S5. The results of the Lsmeans test on the seed box predation model; Table S6. Wheat seed predation model output; Table S7. The results of the Lsmeans test on the wheat seed predation model; Table S8. Mustard seed predation model output; Table S9. The results of the Lsmeans test on the mustard seed predation model; Table S10. Invertebrate and vertebrate predation model outputs; Table S11. The results of the Lsmeans test on the invertebrate and vertebrate predation models.
