# Supplementary material for: Flowering Coriander (Coriandrum sativum) Strips Do Not Enhance Ecosystem Services in Azorean Orchards
Source: Insects. 2023 Jul 14;14(7):634. doi: 10.3390/insects14070634 (PMC10380325; doi:10.3390/insects14070634)
Supplement: Supplementary file 1 [file insects-14-00634-s001.zip › insects-2494211-supplementary.pdf]

Supplementary Material to the article

Flowering coriander (*Coriandrum sativum*) strips do not enhance ecosystem services in Azorean orchards by Marco Ferrante, Gabor L Lövei, Lambert Lavigne, Mario Caballero, Elisa Tarantino, David Horta Lopes, Paulo Monjardino & Paulo AV Borges.

Table S1 Models overview.

Ecological function	Fixed component	Random component	Additional	Distribution
Herbivory	Treatment + Phenology	1|Orchard	zi = ~ 1<br>disp = ~ Treatment + Phenology	beta
Seed box predation	Treatment + Phenology + Seed Type + Box Type	1| Orchard/Replicate		binomial
Wheat seed predation	Treatment * Phenology	1| Orchard/Replicate	zi = ~ 1<br>disp = ~ Treatment + Phenology	beta
Mustard seed predation	Treatment + Phenology	1| Orchard/Replicate	zi = ~ 1<br>disp = ~ Treatment + Phenology	beta
Invertebrate predation	Treatment + Phenology	1| Orchard/Patch		binomial
Vertebrate predation	Treatment + Phenology	1| Orchard/Patch		binomial

Table S2 Herbivory model output.

Conditional model	Variance	SD		
Orchard	1.556e-19	3.945-e10		
	**Estimate**	**SE**	**z-value**	**Significance**
Intercept	-2.96	0.19	-15.8	< 0.001
Treatment (Coriander)	-0.12	0.19	-0.6	0.546
Phenology (Flowering)	-2.05	0.21	-9.7	< 0.001
Phenology (Post-flowering)	-1.22	0.27	-4.53	< 0.001
**Zero-inflation model**				
Intercept	-21.89	6676.55	-0.003	0.997
**Dispersion model**				
Intercept	3.62	0.39	9.22	< 0.001
Treatment (Coriander)	0.19	0.37	0.51	0.608
Phenology (Flowering)	2.09	0.44	4.74	< 0.001
Phenology (Post-flowering)	0.34	0.46	0.75	0.456

Table S3 The results of the Lsmeans test on the herbivory model.

	Estimate	SE	z-ratio	Significance
Pre-flowering vs Flowering	2.05	0.21	9.7	< 0.001
Pre-flowering vs Post-flowering	1.22	0.27	4.53	< 0.001
Flowering vs Post-flowering	-0.83	0.27	-3.1	0.006

Table S4 Seed box predation model output.

Conditional model	Variance	SD		
Replicate: Orchard	0.277	0.526		
Orchard	0.299	0.547		
	**Estimate**	**SE**	**z-value**	**Significance**
Intercept	-0.86	0.5	-1.73	0.084
Treatment (Coriander)	-0.68	0.33	-2.04	0.041
Phenology (Flowering)	-0.54	0.4	-1.34	0.18
Phenology (Post-flowering)	-0.88	0.41	-2.16	0.031
Seed (Wheat)	2.89	0.39	7.51	< 0.001
Box (Open)	0.32	0.30	1.05	0.295

Table S5 The results of the Lsmeans test on the seed box predation model.

Comparison	Estimate	SE	z-ratio	Significance
Pre-flowering vs Flowering	0.54	0.4	1.34	0.372
Pre-flowering vs Post-flowering	0.88	0.41	2.16	0.078
Flowering vs Post-flowering	0.34	0.4	0.85	0.67

Table S6 Wheat seed predation model output.

Conditional model	Variance	SD		
Replicate: Orchard	0.153	0.392		
Orchard	0.265	0.515		
	**Estimate**	**SE**	**z-value**	**Significance**
Intercept	-1.52	0.4	-3.77	< 0.001
Treatment (Coriander)	0.04	0.3	0.13	0.895
Phenology (Flowering)	1.89	0.41	4.6	< 0.001
Phenology (Post-flowering)	1.89	0.4	4.78	< 0.001
Coriander:Flowering	-1.1	0.55	-2.0	0.046
Coriander:Post-flowering	-1.9	0.59	-3.23	0.001
**Zero-inflation model**				
Intercept	-1.17	0.2	-5.98	< 0.001
**Dispersion model**				
Intercept	1.77	0.38	4.72	< 0.001
Treatment (Coriander)	0.74	0.29	2.54	0.011
Phenology (Flowering)	-2.12	0.37	-5.77	< 0.001
Phenology (Post-flowering)	-1.76	0.39	-4.5	< 0.001

Table S7 The results of the Lsmeans test on the wheat seed predation model.

Control-Coriander comparison	Estimate	SE	z-ratio	Significance
Pre-flowering	-0.04	0.3	-0.13	0.8954
Flowering	1.06	0.46	2.29	0.0219
Post-flowering	1.86	0.52	3.59	0.0003

Table S8 Mustard seed predation model output.

**Conditional model**	**Variance**	**SD**		
Replicate: Orchard	1.675e-11	4.092e-06		
Orchard	7.204e-03	8.487e-02		
	**Estimate**	**SE**	**z-value**	**Significance**
Intercept	-2.76	0.21	-13.28	< 0.001
Treatment (Coriander)	-1.11	0.19	-5.77	< 0.001
Phenology (Flowering)	0.9	0.13	6.66	< 0.001
Phenology (Post-flowering)	0.37	0.2	1.87	0.006
**Zero-inflation model**				
Intercept	1.29	0.2	6.38	< 0.001
**Dispersion model**				
Intercept	3.35	0.45	7.5	< 0.001
Treatment (Coriander)	3.76	0.62	6.05	< 0.001
Phenology (Flowering)	-0.21	0.73	-0.28	0.777
Phenology (Post-flowering)	-1.54	0.65	-2.37	0.018

Table S9 The results of the Lsmeans test on the mustard seed predation model.

	**Estimate**	**SE**	**z-ratio**	**Significance**
Pre-flowering vs Flowering	-0.91	0.13	-6.97	< 0.001
Pre-flowering vs Post-flowering	-0.41	0.2	-2.07	0.095
Flowering vs Post-flowering	0.5	0.2	2.45	0.039

Table S10 Invertebrate and vertebrate predation model outputs.

**Invertebrates**				
**Random effects**	**Variance**	**SD**		
Patch:Orchard	4.539e-14	2.13-e07		
Orchard	0.0	0.0		
	**Estimate**	**SE**	**z-value**	**Significance**
Intercept	-4.08	0.64	-6.42	< 0.001
Treatment (Coriander)	0.12	0.49	0.25	0.81
Phenology (Flowering)	-0.05	0.74	-0.06	0.95
Phenology (Post-flowering)	0.57	0.67	0.84	0.4
**Vertebrates**				
**Random effects**	**Variance**	**SD**		
Patch:Orchard	1.713	1.309		
Orchard	0.0	0.0		
	**Estimate**	**SE**	**z-value**	**Significance**
Intercept	-2.33	0.5	-4.7	< 0.001
Treatment (Coriander)	-1.16	0.44	-2.63	0.008
Phenology (Flowering)	-0.07	0.57	-0.13	0.897
Phenology (Post-flowering)	0.15	0.56	0.27	0.79

Table S11 The results of the Lsmeans test on the invertebrate and vertebrate predation models.

**Invertebrate**	**Estimate**	**SE**	**z-ratio**	**Significance**
Pre-flowering vs Flowering	0.05	0.74	0.06	0.998
Pre-flowering vs Post-flowering	-0.57	0.67	-0.84	0.678
Flowering vs Post-flowering	-0.61	0.56	-1.09	0.523
**Vertebrate**	**Estimate**	**SE**	**z-ratio**	**Significance**
Pre-flowering vs Flowering	0.074	0.57	0.13	0.991
Pre-flowering vs Post-flowering	-0.15	0.56	-0.27	0.962
Flowering vs Post-flowering	-0.22	0.49	-0.46	0.891

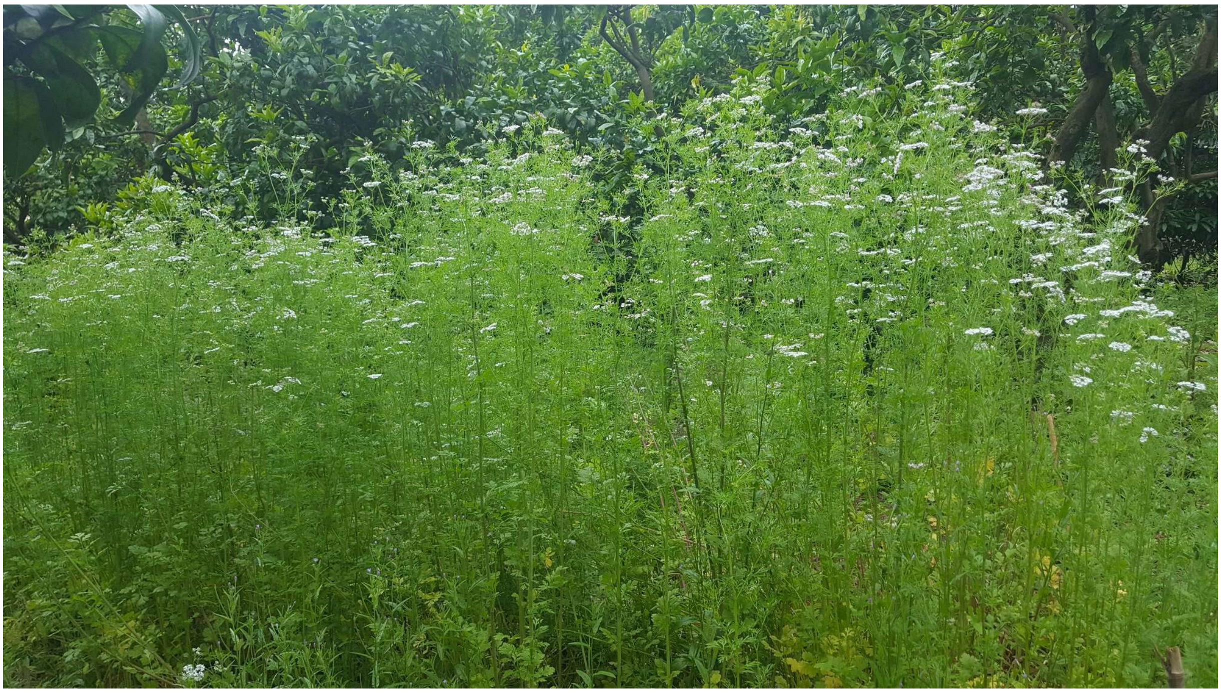

Figure S1. Coriander (*Coriandrum sativum*) strip starting to flower in a mixed orchard on Terceira Island, Azores, Portugal.

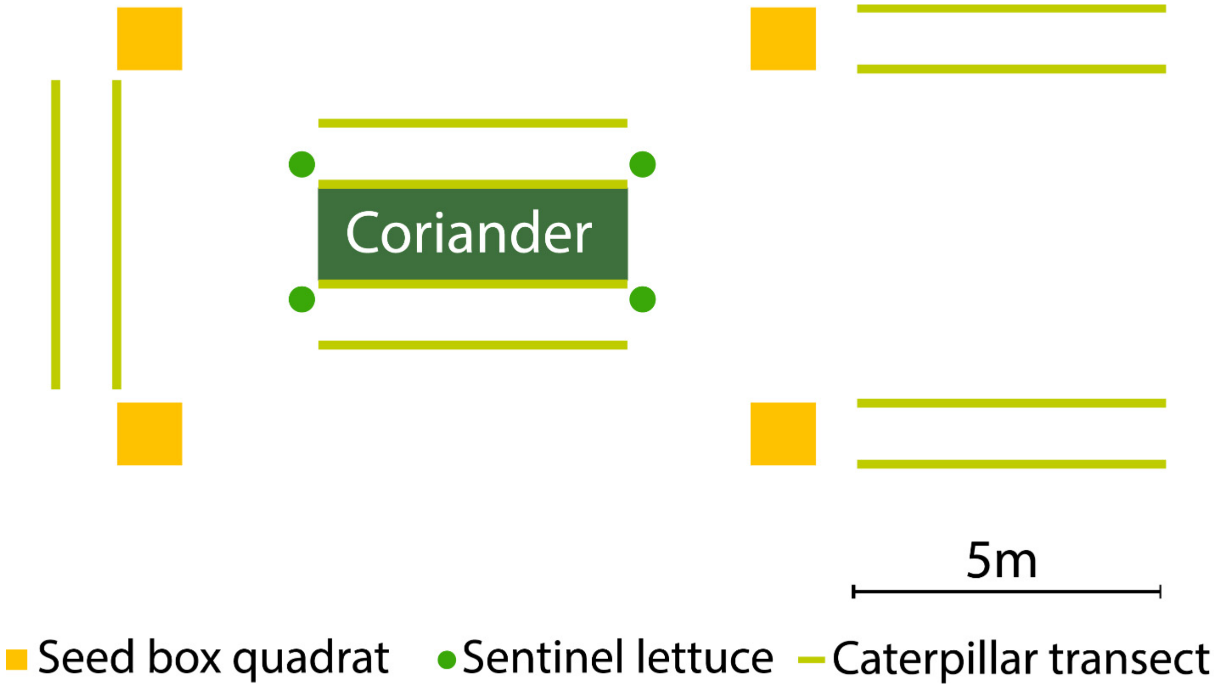

Figure S2. Arrangement of the sentinels in the orchards. The green circles represent individual sentinel lettuce plants; each orange square represents a group of four seed boxes (open and vertebrate exclusion box with mustard seeds, and open and vertebrate exclusion box with wheat grains) arranged in a square, 1 m from each other; the green lines represent a transect consisting of five artificial caterpillars linearly arranged, 1 m from each other.

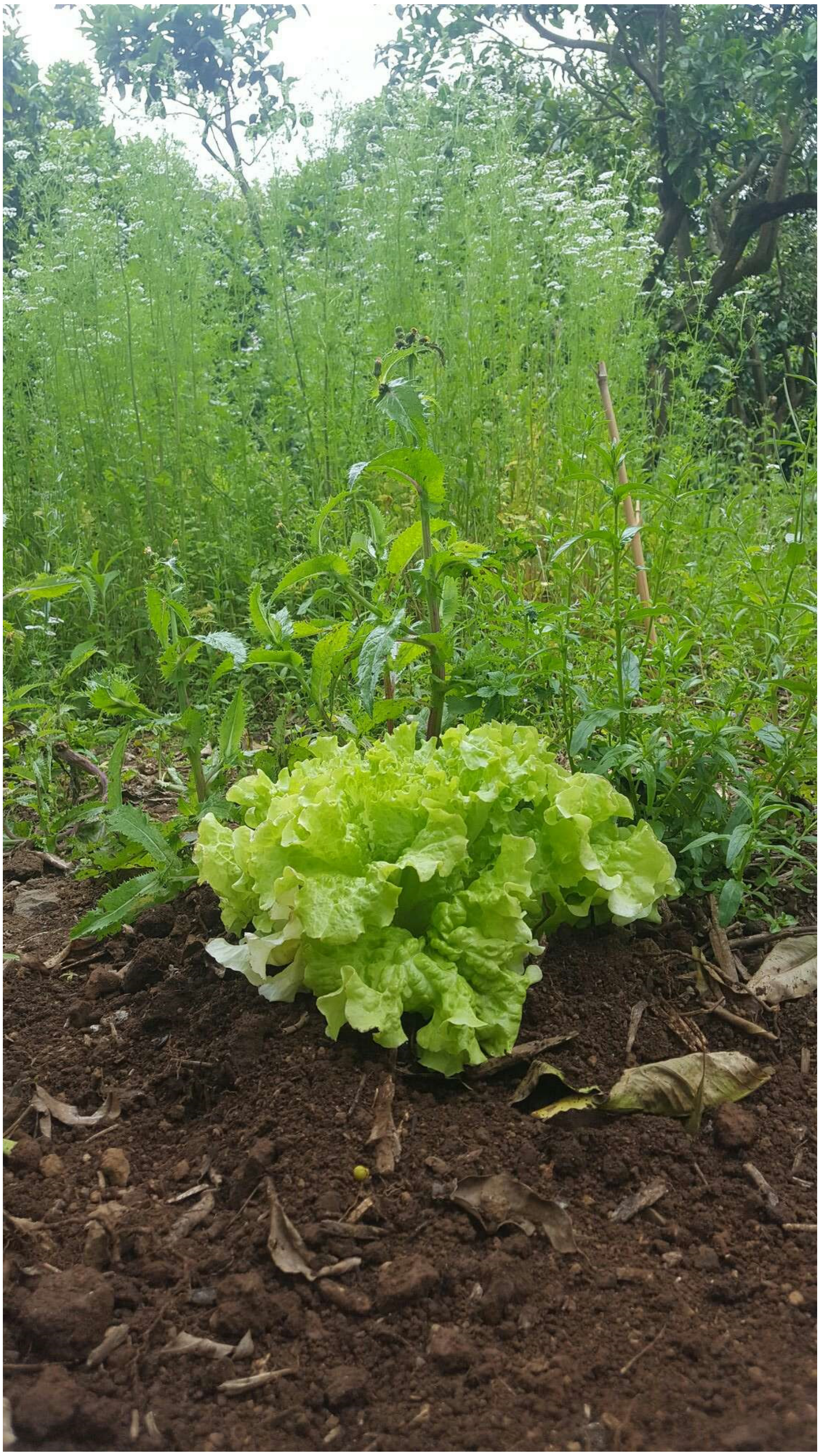

Figure S3. Sentinel lettuce (*Lactuca sativa*) near one of the corners of the coriander strip in a mixed orchard on Terceira Island, Azores, Portugal.

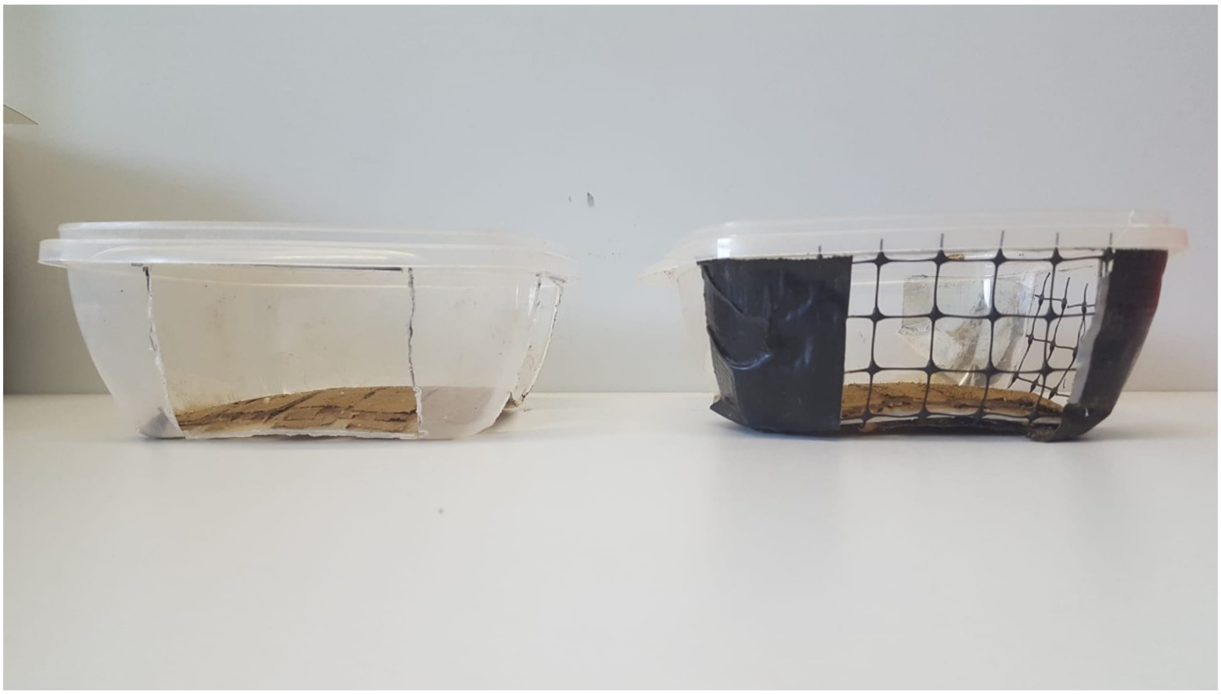

Figure S4. An “open” box accessible to both invertebrates and vertebrates (on the left) and a vertebrate exclusion box (on the right). Seed boxes were covered with a lid to avoid rain and interference by birds.

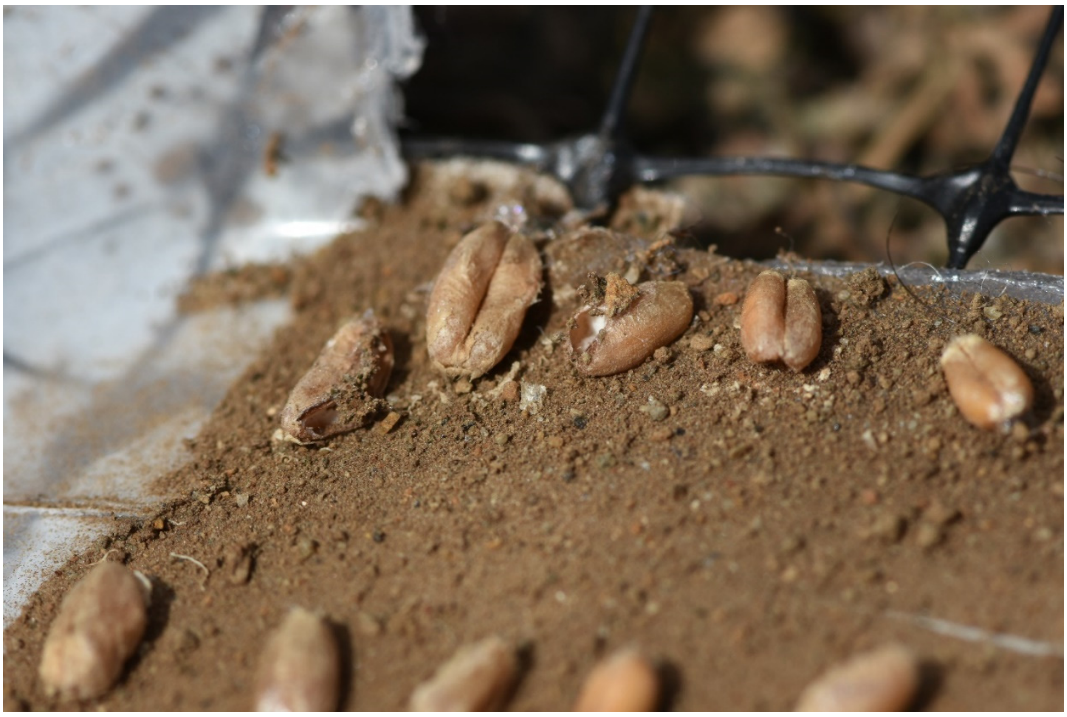

Figure S5. Wheat seeds drilled by seed predators inside a vertebrate exclusion box in a mixed orchard on Terceira Island, Azores, Portugal.

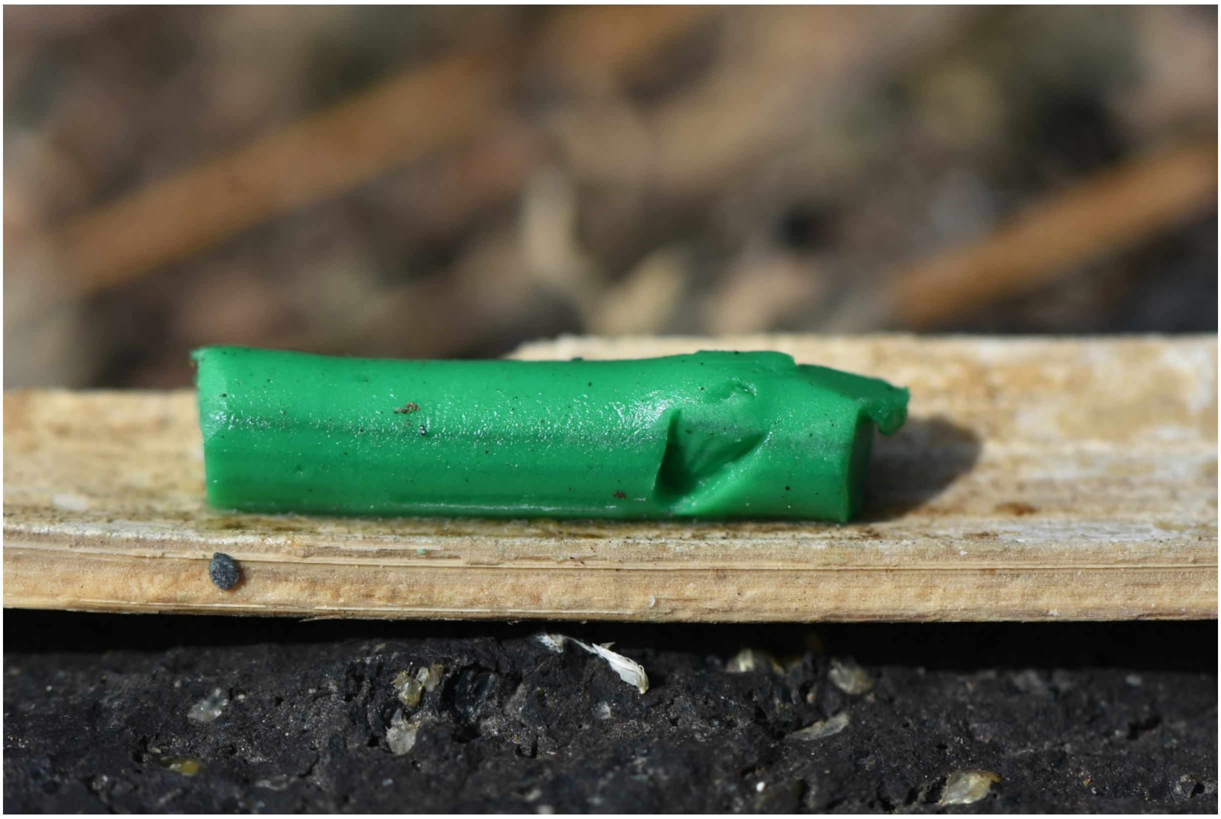

Figure S6. Artificial caterpillar made of plasticine glued to a piece of reed and exposed at ground level in a mixed orchard on Terceira Island, Azores, Portugal. The artificial caterpillar has been attacked by a bird, which can be identified by the typical “v” mark.

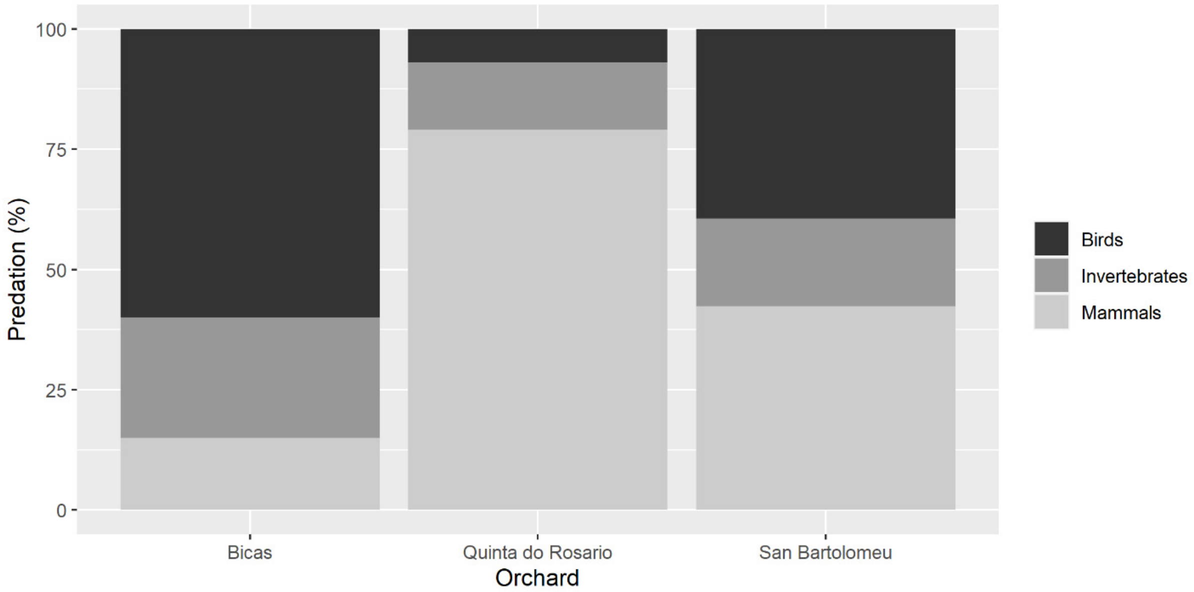

Figure S7. Relative predation by birds, invertebrates, and mammals in three Azorean orchards.
